# Embelin-Induced Apoptosis of Human Prostate Cancer Cells Is Mediated through Modulation of Akt and β-Catenin Signaling

**DOI:** 10.1371/journal.pone.0134760

**Published:** 2015-08-07

**Authors:** Nahee Park, Hyoung Seok Baek, Young-Jin Chun

**Affiliations:** College of Pharmacy, Chung-Ang University, Seoul, 156–756, Korea; Kyung Hee University, REPUBLIC OF KOREA

## Abstract

There is increasing evidence that embelin, an active component of *Embelia ribes*, induces apoptosis in human cancer cells, but the detailed mechanisms are still unclear. Here, we have investigated the effect of embelin on the growth of human prostate cancer cells. Embelin strongly inhibited cell growth especially in human prostate cancer cell lines, including PC3, DU145, LNCaP-LN3 and normal prostate epithelial cell, RWPE-1 compared to breast cancer (MDA-MB-231, MCF-7, and T47D), hepatoma (HepG2, Hep3B, and HuH-7), or choriocarcinoma (JEG-3). We observed that embelin induced apoptosis of PC3 cells in a time-dependent manner correlated with decreased expression of Bcl-2, Bcl-xL, and Mcl-1, increased translocation of Bax into mitochondria, and a reduction in the mitochondrial membrane potential. Furthermore, embelin induced voltage-dependent anion channel (VDAC) 1 expression and oligomerization, which may promote cytochrome *c* and AIF release. Because embelin was able to inhibit Akt activation and cyclooxygenase-2 expression, the effects on Wnt/ β-catenin signaling were determined. Embelin activated glycogen synthase kinase (GSK)-3β by preventing phosphorylation and suppressed β-catenin expression. Attenuation of β-catenin-mediated TCF transcriptional activity and gene transcription, such as cyclin D1, c-myc, and matrix metalloproteinase (MMP)-7, were shown in embelin-treated cells. The changes in β-catenin levels in response to embelin were blocked by lithium chloride, a GSK-3 inhibitor, indicating that embelin may decrease β-catenin expression via GSK-3β activation. Furthermore, exposure of PC3 cells to embelin resulted in a significant decrease in cell migration and invasion. In conclusion, these findings suggest that inhibition of Akt signaling and activation of GSK-3β partially contributes to the pro-apoptotic effect of embelin in prostate cancer cells.

## Introduction

Embelin (2, 5-dihydroxy-3-undecyl-1, 4- benzoquinone), isolated as the active component of the fruit of the *Embelia ribes* Burm (Myrsinaceae), has been used to treat fever and shown to have anti-inflammatory, anti-carcinogenic [1], anti-oxidant [2], anti-convulsant [3], and anti-bacterial activities [4,5]. Embelin is known to be a potent small molecule inhibitor of the X-linked inhibitor of apoptosis protein (XIAP) that abrogates binding of XIAP to procaspase-9 [1]. Embelin acts as a potent inhibitor of NF-*κ*B [6,7] and shows cytotoxic effects on a variety of cancer cell lines [8]. Although embelin is known to suppress cell proliferation and induce apoptosis in many human cancer cells, the molecular mechanisms of these effects remain unclear. Apoptosis plays a major role in controlling cellular integrity and is strictly regulated. Two main distinct apoptotic pathways have been developed, the intrinsic and extrinsic pathways. Initiation signals for the intrinsic pathway are generated by developmental cues or cellular damage that causes the loss of mitochondrial membrane potential and the release of pro-apoptotic proteins [9, 10]. However, the mechanisms by which apoptogenic initiators cross the outer mitochondrial membrane (OMM) have not yet been fully resolved. Voltage-dependent anion channel (VDAC) 1 located in the OMM has been proposed as an important component of the permeability transition pore (PTP), which leads to the release of cytochrome c and apoptosis inducing factor (AIF) [11,12]. Oligomerization of VDAC1 to form a large pore structure in response to several pro-apoptotic signals may be required for cytochrome c release [13]. Moreover, interaction of VDAC1 with Bax, a proapoptotic Bcl-2 family protein can form larger complex than VDAC1 alone or Bax alone and promotes the opening of PTP [14]. However, anti-apoptotic Bcl-2 family proteins such as Bcl-2, Bcl-xL, or Mcl-1 prevent the PTP opening and are associated with treatment resistance and progression in many types of cancer, including prostate cancer [15]. According to previous studies, Mcl-1 is a crucial regulator of apoptosis and differentiation and is overexpressed in the majority of prostate cancer cells [16]. Chen et al. reported a novel pathway, which consists of Akt, cyclooxygenase-2 (COX-2), and Mcl-1, for acquired resistance to apoptosis in cancer cells [17]. Akt regulates cellular signaling networks that are involved in processes linked to the cellular proliferation, differentiation, and metabolism and activates a COX-2-mediated anti-apoptotic pathway that involves Mcl-1 [18,19]. Phosphorylation by Akt at the Ser 9 residue of GSK-3β leads to its inactivation [20, 21], resulting in the stabilization of β-catenin, which is correlated with increased transcription of down-stream target genes [22]. Furthermore, inhibition of GSK-3β phosphorylation clearly suppresses β-catenin expression and resistance to apoptosis. The present study shows that embelin induces mitochondrial-dependent apoptosis and suppression of β-catenin expression via Akt inhibition and GSK-3β activation in human prostate cancer cells.

## Materials and Methods

### Cell culture

Human prostate cancer cell lines PC3, DU145, and LNCaP-LN3, human prostate epithelial cell line RWPE-1, human liver hepatocellular carcinoma cell line HepG2, Hep3B, and HuH-7 or human breast cancer cell line MDA-MB-231, MCF-7, and T47D cells were obtained from the American Type Culture Collection (Manassas, VA) and were cultured in RPMI 1640 supplemented with 10% (v/v) heat-inactivated fetal bovine serum (FBS), 100 U/mL penicillin, and 100 μg/mL streptomycin. The human choriocarcinoma cell line JEG-3 was purchased from the Korean Cell Line Bank and was cultured in DMEM medium with 10% (v/v) heat-inactivated FBS, 100 U/mL penicillin, and 100 μg/mL streptomycin. Cells were maintained at 37°C in a humidified atmosphere of 5% CO_2_.

### Reagents

Embelin was purchased from Sigma-Aldrich (St. Louis, MO). A 100 mM solution of embelin was prepared in dimethyl sulfoxide, stored as small aliquots at -20°C and then diluted as needed in cell culture medium. A 8M solution of lithium chloride was obtained from Sigma-Aldrich and diluted as needed in cell culture medium. FBS and RPMI 1640 medium were purchased from HyClone (Logan, UT). The Neon transfection system, JC-1 assay kit and COX-4 antibody were from Life Technologies (Carlsbad, CA). The bicinchoninic acid (BCA) protein assay kit and ECL kit were from Thermo Scientific (Rockford, IL). Antibodies against phospho-Akt (Ser 473), GSK-3β, phospho-GSK-3β, or Bcl-2 were from Cell Signaling Technology (Beverly, MA). Antibodies against Total-Akt, VDAC1, Bcl-xL, Bax, β-catenin, cyclin D1, GAPDH or goat anti-rabbit IgG-Texas Red and Ultra Cruz mounting medium were from Santa Cruz Biotechnology (Santa Cruz, CA). Cyclooxygenase-2 (COX-2), Mcl-1, cytochrome *c*, AIF, β-catenin, c-myc or histone H1 antibodies were from Millipore Co. (Bedford, MA). All other chemicals were of the highest purity or molecular biology grade and were obtained from commercial sources.

### Cell viability assay

Cells (1 × 10^4^ cells/well) were added to a 96-well round-bottom microplate and were incubated for the designated time at 37°C. After treatment for the designated time, the cells were treated with 10 μL of EZ-CyTox (Daeil Lab Service, Korea) was added to each well. After 2 h incubation at 37°C, the absorbance was measured at 450 nm using a GENios Pro microplate reader (TECAN, Switzerland).

### Annexin V/PI apoptosis assay

Cells were pelleted at 1200 rpm and washed once with 1 mL of ice-cold phosphate buffered saline (PBS). The resulting pellet was resuspended in 1 mL of 1 × binding buffer. The resulting mixture was kept on ice for 60 min, after which the cells were permeabilized with 50 μg/mL annexin V-FITC and 50 μg/mL propidium iodide in 1× binding buffer. The samples were kept at 37°C for 60 min and analyzed immediately using a BD FACScan flow cytometer (Becton Dickinson, San Jose, CA).

### Measurement of mitochondrial membrane potential (Δ*ψ*m)

Cells grown on poly-d-lysine-coated coverslips were treated for 24 h with embelin in growth medium, rapidly washed with PBS, and then labeled with 2 μM JC-1 for 30 min at 37°C in a 5% CO_2_ incubator. After washing several times with PBS, the coverslips were mounted on glass slides using Ultra Cruz mounting medium. Fluorescence signals were analyzed with a LSM 510 META Confocal Laser Scanning Microscope (Zeiss, Jena, Germany).

### Transient transfection

For transient transfection, cells were transfected with pECE myr-Akt or human COX-2 promoter luciferase constructs and pRL-renilla (Promega) vectors using the Neon transfection system as recommended by the manufacturer. Briefly, one day prior to transfection, approximately 5 × 10^5^ cells per 60-mm plate were seeded in RPMI 1640 medium containing 10% FBS. Cells were washed in PBS and then resuspended in Opti-MEM serum-free medium. Transfection was carried out with 2 μg of plasmid DNA for 5 h at 37°C. After transfection, cells were maintained in RPMI medium containing 10% FBS for 48 h.

### Subcellular fractionation

After treatment, cells were harvested and washed with ice-cold PBS. Subcellular fractionation was performed using the Mitochondria Isolation kit for cultured cells and NE-PER Nuclear and Cytoplasmic Extraction kit from Thermo Scientific. Western blotting was carried out using antibodies against the following control marker proteins: GAPDH for the cytosolic fraction, COX-4 for the mitochondrial fraction or histone-H1for the nuclear fraction.

### Western blot analysis

Cells were solubilized with ice-cold lysis buffer (pH 7.4) containing 25 mM HEPES, 1% triton X-100, 50 mM NaCl, 1 mM EDTA, 1 mM EGTA, 1 mM PMSF, and 1 μg/mL leupeptin. The extracted proteins (30 μg) were separated by sodium dodecyl sulfate-polyacrylamide gel electrophoresis (SDS-PAGE) on 10% polyacrylamide gels, and were electrophoretically transferred onto PVDF membranes. Membranes were blocked in 5% (w/v) nonfat dried milk in Tris-buffered saline for 2 h at 4°C and then were incubated overnight with primary antibodies at a 1:1000 dilution in 5% (w/v) Tris-buffered saline containing 0.1% Tween-20. After incubation with secondary antibody for 2 h, proteins were visualized by ECL and the band intensity was analyzed using a ChemiDoc XRS densitometer and quantified by Quantity One software (Bio-Rad, Richmond, CA). Protein concentrations were estimated using the BCA method according to the supplier’s recommendations using bovine serum albumin as a standard.

### Immunofluorescence

Cells grown on poly d-lysine-coated coverslips were treated for 24 h with embelin in growth medium, rapidly washed with PBS, and then treated with growth medium including 100 nM MitoTracker probes (Invitrogen). After 1 h, the cells were fixed with 3.7% (w/v) paraformaldehyde in PBS, pH 7.4, for 30 min at room temperature. After washing with PBS, the cells were blocked for 15 min in PBS containing 5% goat serum and 0.2% Triton X-100, then incubated with primary antibody (1:1000) for 1 h, washed extensively, and stained for 1 h with goat anti-rabbit IgG-Texas Red (1:500). After further washes, the coverslips were mounted on glass slides using Ultra Cruz mounting medium. Fluorescence signals were analyzed by using a LSM 510 META Confocal Laser Scanning Microscope (Carl Zeiss, Germany).

### Cross-linking of VDAC

Following treatment with embelin, sulfo-EGS in DMSO was added to a final concentration of 250 μM. After 25-min incubation at 30°C, the cross-linker was quenched by the addition of 1M Tris-HCl (pH 7.5) to a final concentration of 20 mM. Samples were then solubilized in 1% NP-40 and sonicated for 7 s five times with a 30% pulse using a Vibra-Cell sonicator (Sonics and Materials, Newtown, CT).

### DNA fragmentation assay

DNA fragments were extracted with the Exgene Cell SV genomic DNA purification system (GeneAll, Korea) according to the manufacturer’s protocol. The DNA fragments were separated using gel electrophoresis on a 1% agarose gel. Loaded DNA was visualized by ChemiDoc XRS (Bio Rad).

### Dual luciferase reporter assay

Cells (1×10^6^ cells/well) were cotransfected with 2 μg of human COX-2 promoter luciferase constructs and pRL-renilla (Promega) vectors or 2 μg of TOPFlash luciferase vectors with the wild-type TCF binding sites and FOPFlash luciferase vector with the mutant TCF binding sites according to the manufacturer’s protocol using the Neon transfection system. After 48 h, cells were treated with 30 μM embelin for the indicated times, and lysed with passive lysis buffer, and luciferase activities were measured consecutively using the Dual Luciferase Assay System (Promega) with a FilterMax F3 microplate reader (Molecular Devices, CA).

### Wound healing assay

Cells (1 × 10^6^ cells/well) were seeded into 6-well cell culture plates. Cells were allowed to grow to confluency in RPMI1640 medium containing 10% FBS. Cells were washed with PBS and incubated with 25 μg/mL of mitomycin C for 30min. A1-mm-wide scratch was made across the cell layer using a sterile pipette tip. Plates were photographed after the indicated time.

### Invasion assay

Cell invasion was measured using the cell invasion assay kit (Chemicon, Temecula, CA) according to the manufacturer's instructions. Briefly, cells were seeded on an invasion chamber insert containing an 8 μm pore size polycarbonate membrane coated with a thin layer of polymerized collagen. Invading cells on the bottom of the insert membrane were stained and photographed.

### Gelatin Zymography

Conditioned media were analyzed for gelatinases by gelatin zymography. For gelatinases, samples were separated under non-reducing conditions on 10% SDS polyacrylamide gels incorporating 0.1% gelatin. After SDS-PAGE, the gel was incubated with renaturation buffer for 15 min three times. Then, the gels were replaced in development buffer at 37°C overnight. The gels were washed and photographed after staining with 0.5% Coomassie blue solution for 30 min.

### Statistical analysis

Statistical analysis was performed using one-way analysis of variance, followed by Dunnett’s pairwise multiple comparison t-test with GraphPad Prism software (GraphPad Software Inc., CA) when appropriate. Differences were considered statistically significant at *p< 0.05.

## Results

### Embelin inhibits proliferation of human prostate cancer cells

**Fig 1:**
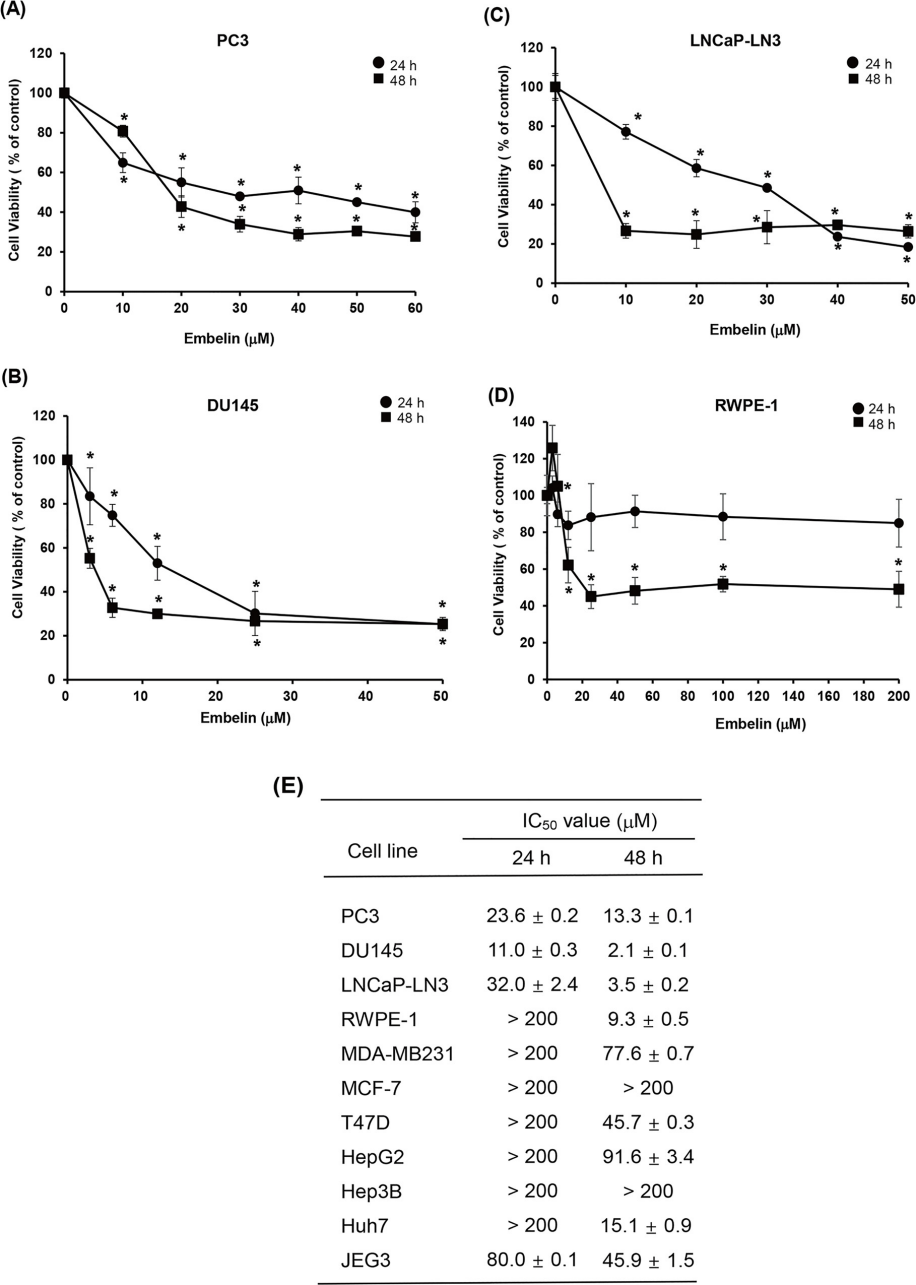
Embelin inhibits the cell growth of human prostate cancer cell lines in a concentration- and time-dependent manner. Cells were treated with embelin at various concentrations for the indicated times. Cell viability was measured by CCK. Formazan formation was quantified by spectrophotometry at 450 nm. The percentage of cells surviving in each group relative to the control was calculated. (A) PC3 cell. (B) DU145 cell. (C) LNCaP-LN3 cell. (D) RWPE-1 cell. (E) IC_50_ values of embelin in various human cancer cell lines. The IC_50_ values were analysed using GraphPad Prism software. Values represent mean ± S.D. of three independent determinations. *, significantly different from the untreated control cells (*p*< 0.05).

Various human cancer cell lines, including prostate cancer cells (PC3, DU145 and LNCaP-LN3), human prostate epithelial cell (RWPE-1), breast cancer cells (MDA-MB-231, MCF-7, and T47D), hepatoma cells (HepG2, Hep3B, and HuH-7), and choriocarcinoma cells (JEG-3) were treated with various concentrations of embelin to determine the effects of embelin on cellular proliferation (Fig 1). Embelin significantly inhibited the growth of human prostate cancer cells in comparison with its effects on other cancer cells. Cell viability was decreased by embelin in human prostate cancer cell lines and prostate epithelial cell including PC3, DU145, LNCaP-LN3 and RWPE-1 with IC_50_ values of 23.6 μM, 11.0 μM, 32.0 μM, and over 200 μM respectively when cells were cultured for 24 h (Fig 1E). Prostate cancer cell lines were clearly inhibited by embelin, but normal prostate cell was not affected by embelin in low concentration for 24 h. For further studies, 30 μM concentration of embelin was selected for treatment of PC3 cells.

### Induction of apoptosis by embelin

**Fig 2:**
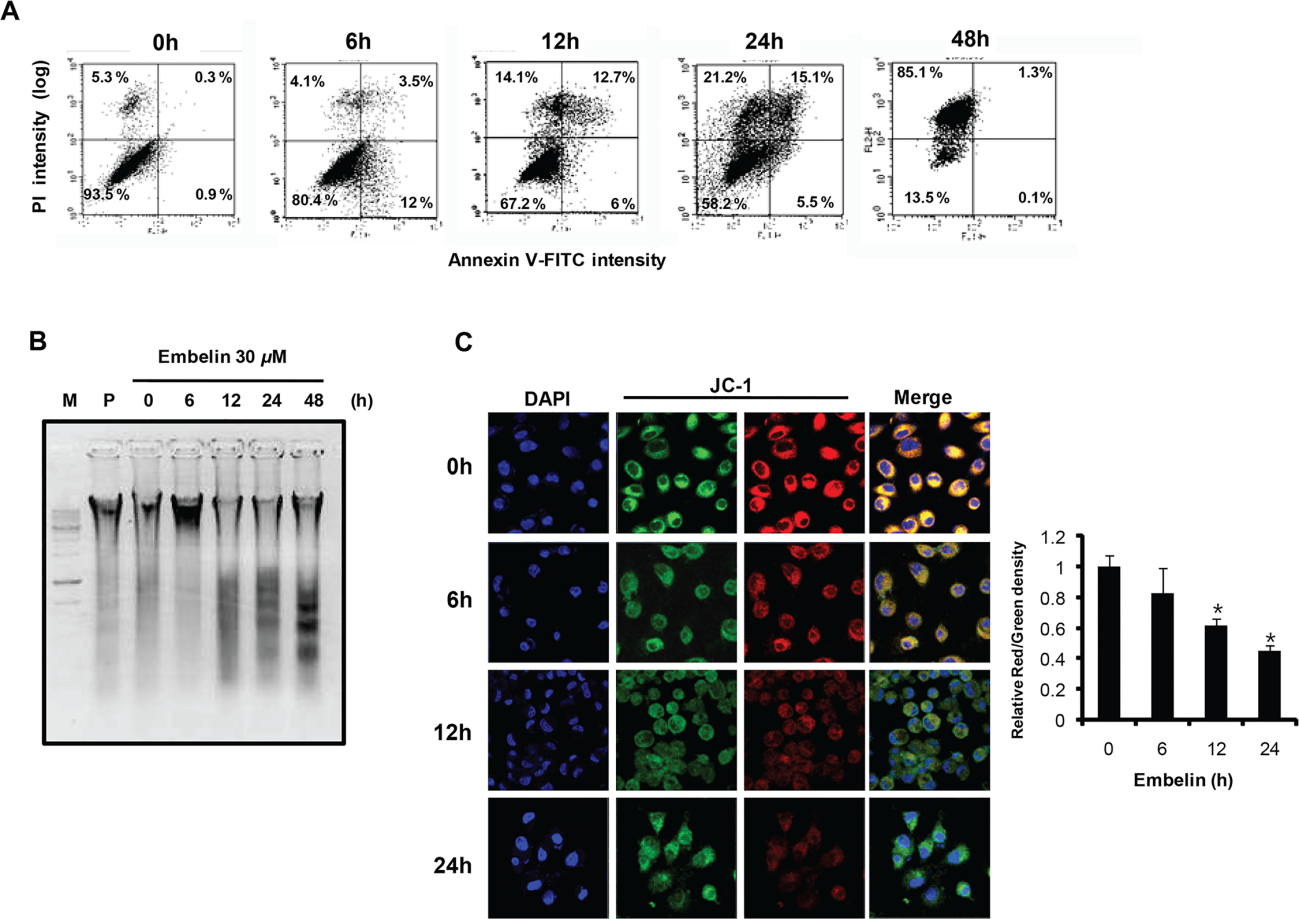
Embelin induces mitochondrial apoptosis in PC3 cells. (A) Cells were incubated for the indicated time periods with embelin (30 μM), and were stained with annexin V-FITC and PI. Apoptosis was measured by flow cytometry. The cell populations were discriminated in each quadrant as viable cells in the lower left (annexin V negative/PI negative), early apoptotic cells in the upper left (annexin V positive/PI negative), late apoptotic cells in the upper right (annexin V positive/PI positive), and necrotic cells in the lower right quadrant (annexin V negative/PI positive). (B) Induction of DNA fragmentation in PC3 cells by embelin. Cells were incubated for the indicated time periods with embelin. Chromosomal DNA was isolated and DNA fragmentation was determined. M, DNA marker; P, paclitaxel (1 μM). (C) After PC3 cells were incubated with embelin for time periods, cells were labeled with 2 M JC-1 for 30 min and Δ*ψ*_m_ was determined by confocal microscopy. The ratio of JC-1 aggregate (red) to monomer (green) intensity was calculated with Image J software. Values represent mean ± S.D. of three independent determinations. *, significantly different from the untreated control cells (*p*< 0.05).

To elucidate whether embelin induces apoptosis in PC3 cells, flow cytometric analysis was performed with annexin V- and propidium iodide (PI)-stained cells. Treatment with 30 μM embelin for up to 48 h caused a strong increase in the rate of apoptosis. Cells treated with 30 μM embelin for 12 h and 24 h showed a 15.6-fold and 17.2-fold increase in apoptosis compared to untreated cells (Fig 2A). After 48 h incubation, a significant increase in necrosis was observed in embelin-treated cells. Treatment with embelin also induced chromosomal DNA fragmentation in a time-dependent manner (Fig 2B). Because the mitochondrial permeability transition may lead to apoptosis, we determined the effect of embelin on mitochondrial membrane potential (Δ*ψ*_m_). Fig 2C showed that embelin strongly decreased Δ*ψ*_m_, indicated as the red/green fluorescence ratio in a time-dependent manner. These results suggest that embelin is able to induce apoptosis by changing mitochondrial membrane permeability.

**Fig 3:**
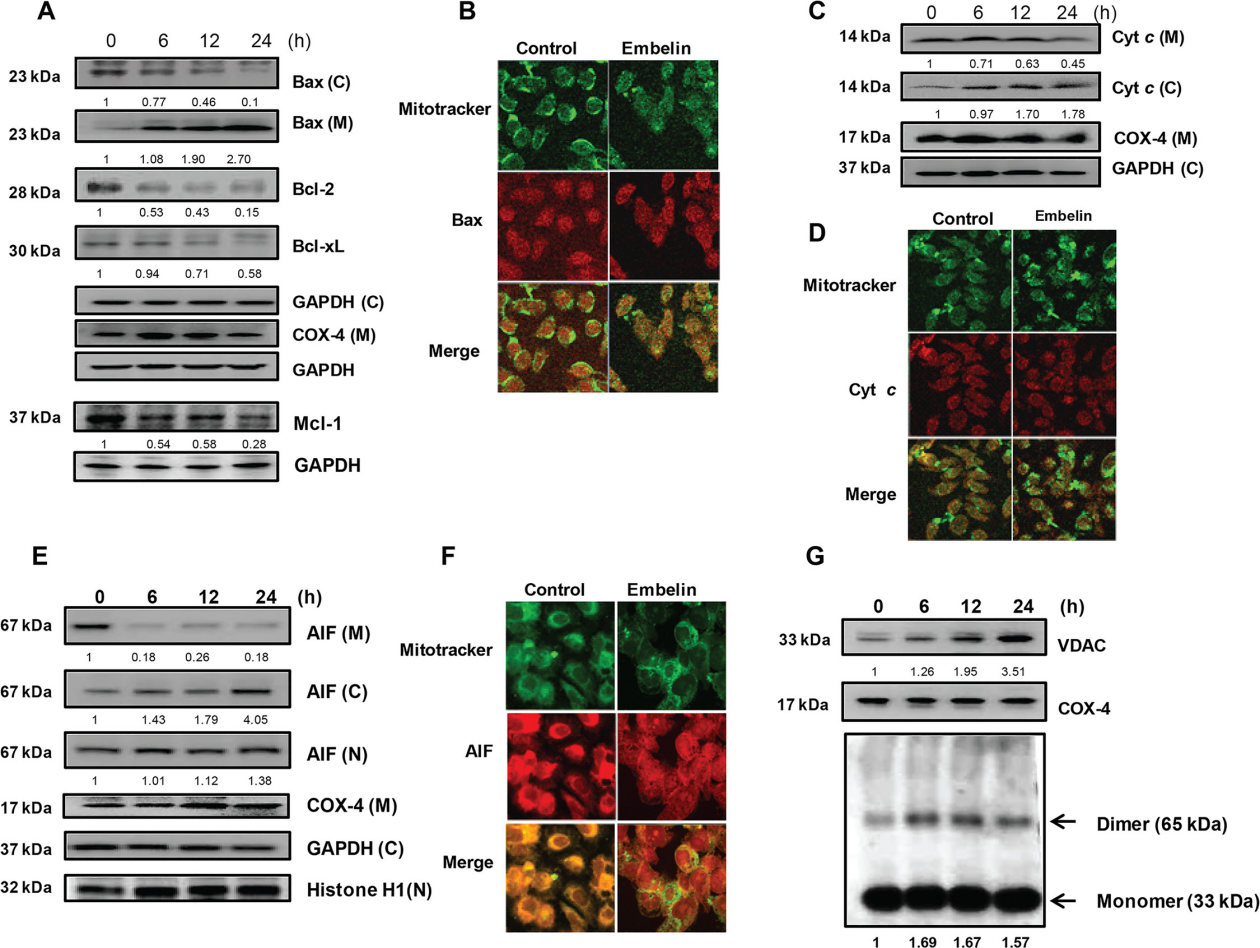
Embelin induces pro-apoptotic proteins and suppresses anti-apoptotic proteins in PC3 cells. (A), (C), (E) Translocation of pro-apoptotic protein (Bax), cytochrome *c*, AIF, and level of anti-apoptotic proteins (Bcl-2, Bcl-xL, Mcl-1) were analyzed by western blotting. Cells were treated with embelin for the indicated time periods. After incubation, cells were harvested and the cytosolic and mitochondrial fractions were isolated. Extracted proteins were resolved by SDS-PAGE (10%) and Western blot analysis was conducted. GAPDH level was determined as loading controls for cytosol and total lysates. COX-4 level was determined as a loading control for mitochondria. Histone H1 level was determined as a loading control for nuclear fraction. C, cytosolic fraction, M, mitochondrial fraction, N, nuclear fraction. The numbers between the blots are the ratios of the intensity of bands after normalized with control. (B), (D), (F) Translocation of Bax (B), cytochrome *c* (D), and AIF (F). Cells were cultured on microscopic slides and treated with embelin for 24 h. After treatment, cells were fixed, permeabilized, and subsequently stained using specific antibody. Cells were then stained with the Texas-Red-labeled secondary antibody. Fluorescence was determined using confocal microscopy. G, Expression of VDAC1 and oligomerization of VDAC1. Embelin-treated cells were harvested and then incubated with sulfo-EGS (250 μM) for 25 min at 30°C. After proteins were resolved by SDS-PAGE (8%), VDAC1 proteins were measured using Western blot analysis. A 33 kDa band represents VDAC1 monomers while a band at 65 kDa represents the VDAC1 dimer. Mitochondrial fraction was isolated and resolved by SDS-PAGE (12%) and Western blot analysis was conducted. COX-4 level was determined as a loading control for mitochondria.

To determine whether embelin affects the levels of apoptosis-related proteins in mitochondria, we measured the amount of Bax (pro-apoptotic), Bcl-2, Bcl-xL, and Mcl-1 (anti-apoptotic). As shown in Fig 3A and 3B, we found that embelin (30 μM) strongly induced translocation of Bax from cytosol to mitochondria. The expression levels of anti-apoptotic Bcl-2 family proteins such as Bcl-2, Bcl-xL, and Mcl-1 were significantly decreased by embelin. At 24 h after embelin treatment, the levels of Bcl-2, Bcl-xL, and Mcl-1 were 15%, 58%, and 28% of the control level, respectively. Release of cytochrome *c* from mitochondria to cytosol was also enhanced in the presence of embelin (Fig 3C). At 24 h after embelin treatment, the cytochrome *c* level was decreased to 45% in mitochondria, but in the cytosol cytochrome *c* level was increased to 1.8-fold of the control level. Confocal microscopic analysis also showed that embelin enhances Bax translocation to the mitochondria and cytochrome *c* release to the cytosol (Fig 3B and 3D). We also found that embelin induces translocation of apoptosis inducing factor (AIF) from the mitochondria, through the cytosol, and finally to the nucleus (Fig 3E). Confocal microscopic analysis indicated that treatment with embelin enhances AIF translocation to the nucleus (Fig 3F). To determine whether embelin induces oligomerization of VDAC to promote changes in Δ*ψ*_m_, and release of cytochrome *c* and AIF, cells were treated with sulfo-EGS to generate cross-linking between VDAC, and oligomerization of VDAC was determined by Western blotting using an anti-VDAC1 antibody. When cells were treated with embelin (30 μM) for up to 24 h, embelin clearly induced expression and dimerization of VDAC1 in a time-dependent manner (Fig 3G). These results suggest that VDAC1 could be a mediator of embelin-induced apoptosis and that VDAC oligomerization induced by embelin could potentially determine its gating capacity for the efflux of mitochondrial proteins, such as cytochrome *c* and AIF.

### Inhibition by embelin of Akt activation and β-catenin pathway

**Fig 4:**
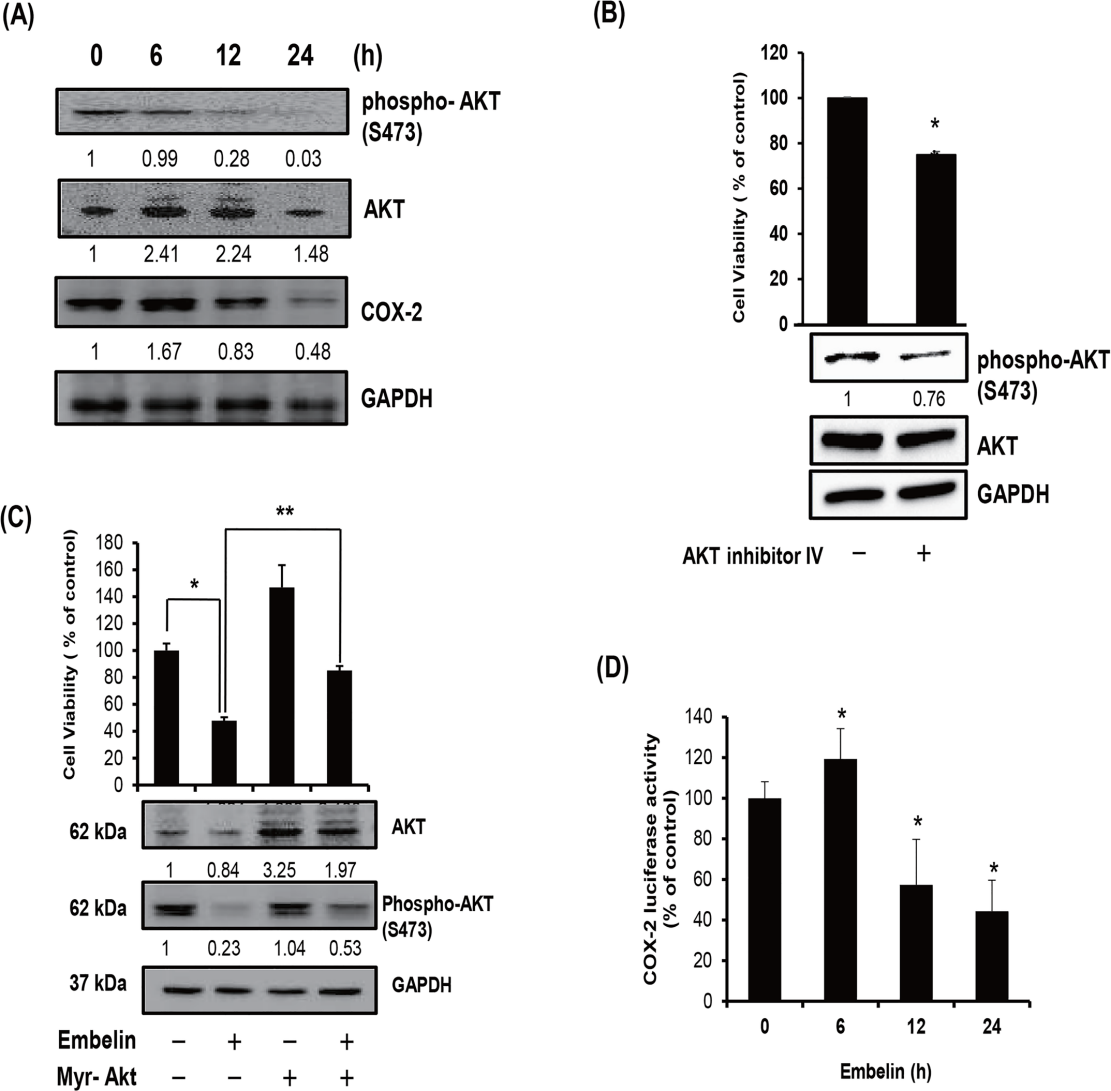
Inhibition of Akt and COX-2 expression by embelin in PC3 cells. (A) Cells were treated with 30 μM embelin for the indicated time periods, and whole cell lysates were prepared, and extracted proteins were resolved by SDS-PAGE (10%) and Western blot analysis using phospho-Akt, total Akt, and COX-2 antibodies was conducted. The numbers between the blots are the ratios of the intensity of bands after normalized with control. (B) Cells were transfected with Myr-Akt plasmid and then treated with 30 μM of embelin for 24h. Cell viability was measured by CCK. Values represent mean ± S.D. of three independent determinations. *, significantly different from the untreated control cells (*p*< 0.01) and **, significantly different from the embelin only-treated cells (*p*<0.001). Whole cell lysates were prepared and the levels of total Akt and phospho-Akt were determined by Western blot analysis. (C) AKT inhibitor IV treated cell lysates were prepared and extracted proteins were resolved by SDS-PAGE (10%) and Western blot analysis using phosphor-Akt, total Akt, and GAPDH antibodies was conducted. Cells were treated with 0.312 μM of AKT inhibitor IV for 24 h. Cell viability was measured by CCK. Formazan formation was quantified by spectrophotometry at 450 nm. The percentage of cells surviving in each group relative to the control was calculated. (D) Cells were transfected with COX-2 luciferase vector and renilla luciferase vector for 48 h. Cells were subjected to the dual-luciferase assay. The relative firefly luciferase activity, normalized by the renilla luciferase activity, is shown. Values represent mean ± S.D. of three independent determinations.*, significantly different from the untreated control (*p*< 0.05).

Previously Chen et al. reported a novel pathway that consists of Akt, and COX-2 for acquired apoptosis resistance in cancer cells [17]. Because we found that embelin suppresses Akt phosphorylation and COX-2 expression were determined. Cells were treated with 30 μM embelin for 6, 12, or 24 h and the levels of phospho-Akt (Ser 473), total Akt, and COX-2 were measured by Western blot analysis. As shown in Fig 4A, phosphorylation of Akt on Ser 473 and expression of COX-2 were significantly decreased by embelin, although the total Akt levels did not change significantly. At 24 h after embelin treatment, the phospho-Akt and COX-2 levels decreased by 99% and 52%, respectively, from the level of control cells. Concomitantly, we evaluated inhibition of Akt activation in PC3 cells, phosphorylation of Akt and cell viability was decreased by Akt inhibitor IV (0.3 μM) (Fig 4B). When cells were transfected with pECE-Myr-Akt plasmid for expression of constitutively active Akt, embelin-mediated decrease of Akt phosphorylation on Ser 473 (Fig 4C). Moreover, we found that the embelin-mediated decrease in cell viability was prevented by myristoylated Akt expression. Embelin also inhibited COX-2 promoter activity, as determined by luciferase reporter assay, indicating that embelin may inhibit Mcl-1 expression through blocking of Akt-COX-Mcl-1 pathway.

**Fig 5:**
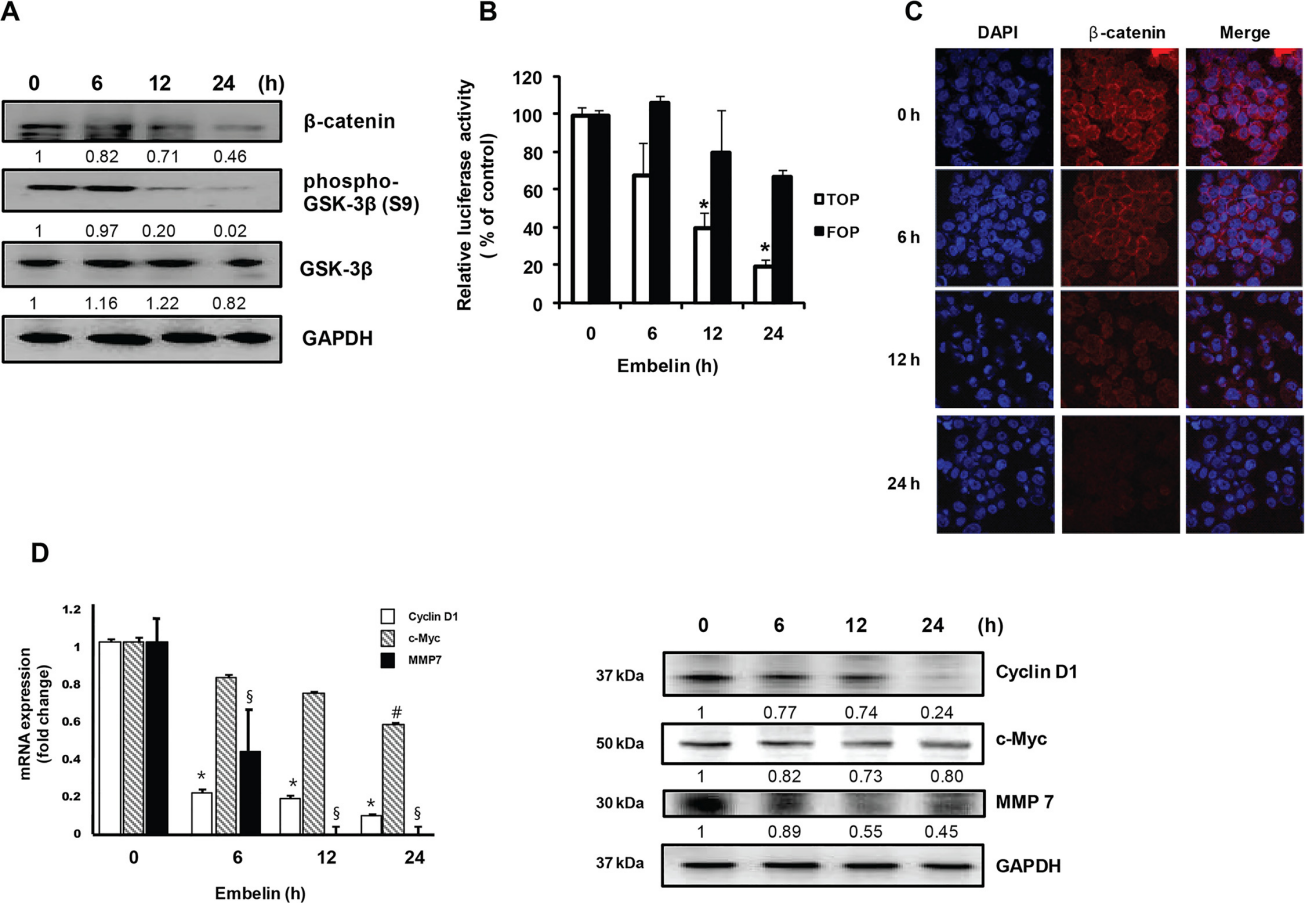
Inhibition of β-catenin expression by embelin. (A) Whole cell lysates were prepared and extracted proteins were resolved by SDS-PAGE (10%) and Western blot analysis using β-catenin, phospho-β-catenin, or GSK-3β antibodies was conducted. The numbers between the blots are the ratios of the intensity of bands after normalized with control. (B) Luciferase assay. TOPFlash or FOPFlash-transfected cells were treated with embelin. The luciferase activity was measured and the relative firefly luciferase activity, normalized by the renilla luciferase activity, is shown. (C) Cells were seeded on cover slip for 24 h and treated with 30 μM embelin up to 24 h. Then the cells were stained with β-catenin antibody and fluorescence was determined using confocal microscopy. (D) Expression of cyclin D1, c-Myc and MMP-7 was determined by using quantitative PCR and Western blot analysis. Values represent mean ± S.D. of three independent determinations.*, ^**#**^ **,** ^**§**^, significantly different from the untreated control (*p*< 0.05). The numbers between the blots are the ratios of the intensity of bands after normalized with control.

Previous report suggests that β-catenin play a crucial role in multiple growth signals in human prostate cancer cells [23]. To determine the effect of embelin on β-catenin expression, PC3 cells were treated with embelin (30 μM) for up to 24 h and Western blotting was performed. Fig 5A showed that embelin is able to decrease the β-catenin level in a time-dependent manner. At 24 h after embelin treatment, the level of β-catenin was decreased by 40% from the level in control cells. We determined TOP flash luciferase activity to measure the level of β-catenin nuclear translocation and TCF transcriptional activation. Embelin (30 μM) significantly inhibited TOPflash activity to 19% of control at 24 h treatment (Fig 5B). Confocal microscopic analysis also confirmed that treatment with embelin clearly decreased the level of β-catenin in PC3 cells (Fig 5C). Transcription of the target genes of β-catenin, such as cyclin D1, c-myc, or MMP-7 was also significantly suppressed by embelin (Fig 5D). Interestingly, mRNA transcription of MMP-7 was almost completely blocked after 12 h treatment with embelin. Western blot analysis also showed that embelin strongly decreased cyclin D1, c-Myc, and MMP-7 protein levels (Fig 5D).

**Fig 6:**
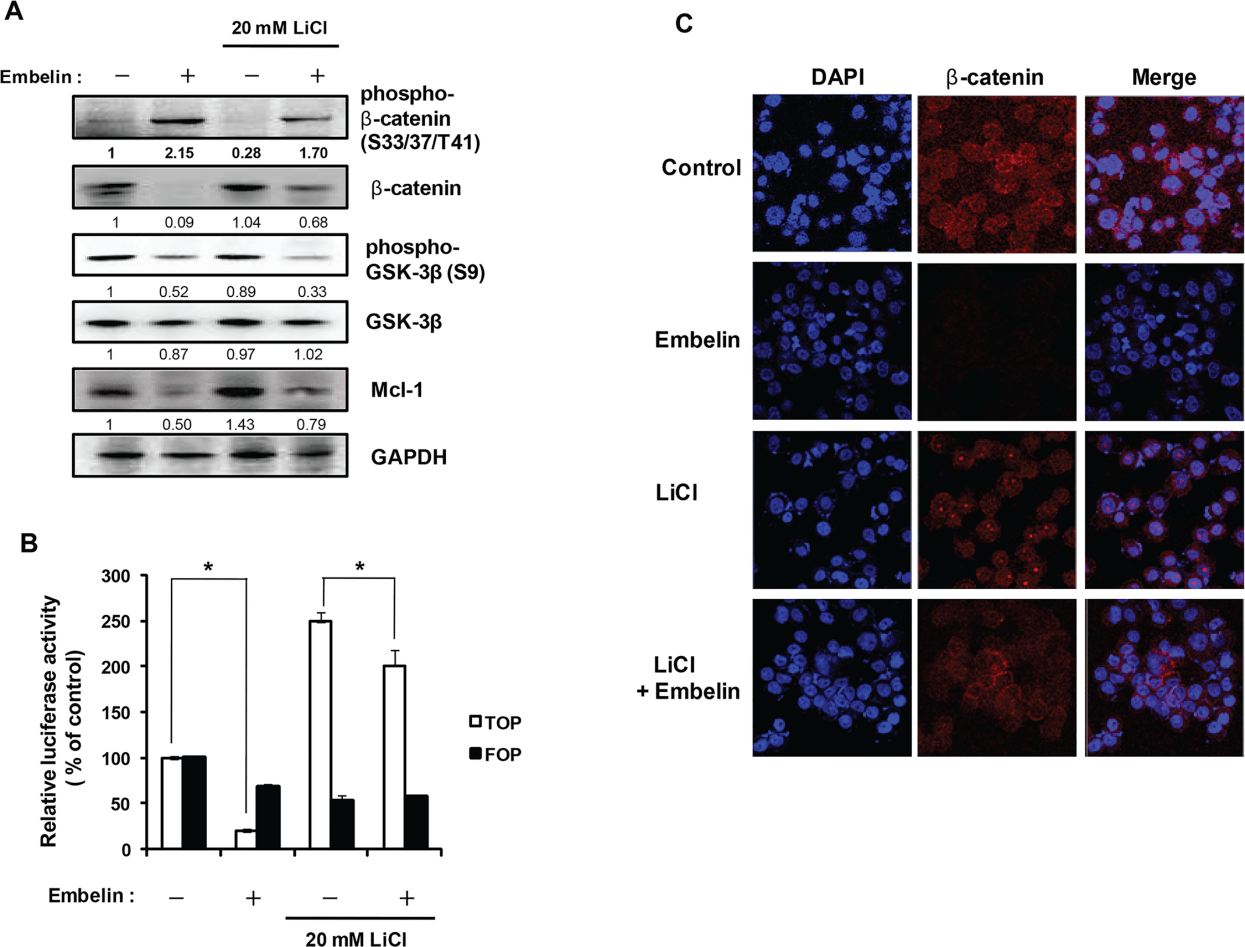
GSK-3β inhibitor recovers embelin-mediated β-catenin downregulation. Embelin- treated cells were co-treated with LiCl 20 mM for 24 h. (A) Whole cell lysate were prepared, and extracted proteins were resolved by SDS-PAGE (10%) and Western blot analysis was conducted. GAPDH level was determined as a loading control. The numbers between the blots are the ratios of the intensity of bands after normalized with control. (B) TOPFlash or FOPFlash-transfected cells were treated with embelin. The luciferase activity was measured and the relative firefly luciferase activity, normalized by the renilla luciferase activity, is shown. (C) Cells were seeded on cover slip for 24 h and treated with 30 μM embelin for 24 h. Cells were stained with β-catenin antibody and fluorescence was determined using confocal microscopy.

Previous report suggested that GSK-3β modulates β-catenin levels by phosphorylation of β-catenin on Ser 33/37 and Thr 41 [24]. We examined whether embelin is able to enhance GSK-3β stability and activity. As shown in Fig 6A, we found that embelin strongly prevented the phosphorylation of GSK-3β on Ser 9 by Akt activation. Embelin strongly induced phosphorylation of β-catenin on Ser 33/37/Thr 41 and might promote β-catenin degradation. We hypothesized that the embelin-mediated increase in phosphorylation of β-catenin may be caused by GSK-3β activation because the decreased β-catenin level by embelin treatment was almost completely recovered by treatment with LiCl (20 mM), a GSK-3β inhibitor. LiCl also decreased the embelin-mediated induction of β-catenin phosphorylation. As treatment with LiCl did not recover embelin-mediated decrease in GSK-3β phosphorylation, LiCl may inhibit GSK-3β activity but not change GSK-3β phosphorylation. Interestingly, the Mcl-1 level was increased 1.4-fold by LiCl treatment and decreased level of Mcl-1by embelin was significantly recovered by LiCl (Fig 6A). These results suggest that Mcl-1 expression is modulated by GSK-3β activity. Maurer et al. reported that inhibition of GSK-3β through Akt activation induced Mcl-1 expression [25]. Our data also confirmed that GSK-3β activation by embelin-mediated Akt inhibition suppresses Mcl-1 expression. Moreover, LiCl induced TOPFlash activity 2.5-fold of control and embelin inhibited the TCF transcriptional activation caused by the LiCl-induced increase in β-catenin (Fig 6B). Confocal microscopic analysis showed that treatment with embelin almost completely inhibited β-catenin expression (Fig 6C). Treatment with LiCl recovered embelin-mediated decrease in β-catenin expression. Interestingly, we found that LiCl strongly induces nuclear translocation and nuclear localization of β-catenin, which may cause induction of β-catenin/TCF transcription in Fig 6B.

### Embelin inhibits PC3 cell migration and invasion

**Fig 7:**
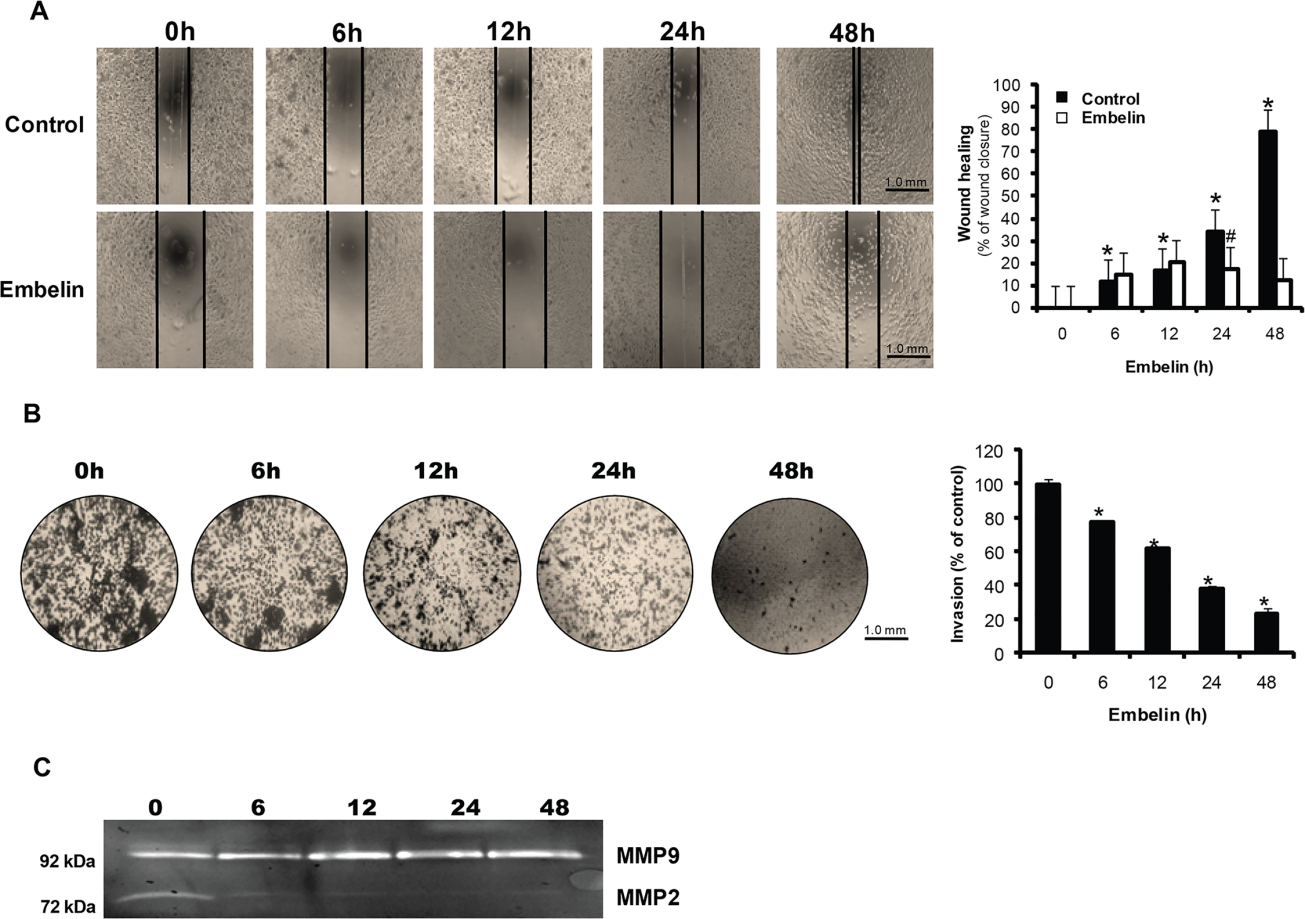
Inhibition of cell migration and invasion by embelin in PC3 cells. (A) Cells were treated with 30 μM embelin in 6-well cell culture plate. A1-mm wide scratch was made across the cell layer using a sterile pipette tip. Each well was photographed at indicated time periods. Black lines on images portray the location of the cell front. The rate of cell migration was determined after estimation of the gap migrated at each time point. Values represent mean ± S.D. of three independent determinations.*, significantly different from the untreated control (*p*< 0.05). ^**#**^ **,** significantly different from the untreated control (*p*< 0.05). (B) Cells were cultured in the upper chamber and incubated with embelin 30 μM for 24 h. The invading cells were fixed and stained, and cells that invaded the lower area of the membrane were photographed. Stained cells were destained with 30% acetic acid for 30 min and determined using microplate reader on 595 nm. The results were plotted and shown in the graph. Values represent mean ± S.D. of three independent determinations. (C) Cells were treated with embelin for 24 h, and conditioned media were harvested. After SDS-PAGE running gel including 0.1% gelatin, the gel was stained with coomassie blue solution and washed by destaining solution. The gel was photographed using ChemiDoc XRS.

To elucidate whether embelin is able to block cancer cell migration, the rate of wound healing was measured in PC3 cells (Fig 7A). At 48 h after embelin treatment, wound healing was inhibited to 66% of control. We also determined the effect of embelin on cancer cell invasion using the transwell invasion assay. Embelin inhibited cancer cell invasion in a time-dependent manner (Fig 7B). At 48 h after embelin treatment, cell invasion was decreased to 23% of control cells. Proteolytic degradation of components of the basement membrane and the extracellular matrix are essential for tissue invasion. Although proteolytic enzymes have been implicated in tumor invasion and metastasis, the MMPs have been the subject of most extensive research [26]. To examine the effect of embelin on activities of MMP-2 and MMP-9 in culture medium, gelatin zymography was performed. Fig 7C showed that embelin strongly inhibited MMP-2 activity while MMP-9 activity was not significantly changed.

## Discussion

Embelin, isolated as the active component of the fruit of *Embelia ribes* Burm, is one of the important small molecular inhibitors of XIAP that has been identified using computational screening from natural product library [1]. Previously, embelin is known to suppress cell proliferation and to induce apoptosis in various human cancer cells [8, 27, 28]. Consistent with previous results, in this study, we showed that embelin is able to inhibit cancer cell growth through induction of apoptosis. The cytotoxic effects of embelin were greater in human prostate cancer cell lines than the other cell lines or prostate epithelial cells, although the reasons for this difference are not completely understood.

To elucidate the molecular mechanism of embelin-induced apoptosis in human prostate cancer cells, we first demonstrated that embelin caused a mitochondrial-dependent apoptosis through a reduction in mitochondrial membrane potential and a release of apoptogenic factors such as cytochrome *c* and AIF. We found that embelin produced a significant translocation of Bax from the cytosol to mitochondria via VDAC1, a pro-apoptotic protein that mediates the exchange of metabolites and energy substrates between the cytosol and the mitochondria [29, 30, 31]. These data suggest that changes in VDAC1 levels in mitochondria may enhance channel formation in the OMM leading to a reduced Δ*ψ*_m_. Our data clearly showed that VDAC1 expression and oligomerization were significantly increased in mitochondria of embelin-treated cells. Previously, Strasser et al. suggested that VDAC1 may act as an activator of Bax and Bax/VDAC1 interaction is able to displace anti-apoptotic proteins, such as Bcl-2 [32], allowing Bax oligomerization and facilitating a reduction of the mitochondrial membrane potential and subsequent apoptosis. Although we did not measure Bax oligomerization, an increase in Bax and VDAC1 levels in mitochondria by embelin may promote oligomerization of both proteins to induce apoptosis.

Embelin also inhibits expression of anti-apoptotic proteins such as Bcl-2, Bcl-xL, and Mcl-1. Chen et al. reported that activation of Akt and induction of COX-2 and Mcl-1 expression may be needed for acquired apoptosis resistance in cancer cells [17]. Our data strongly suggest that embelin prevents Akt activation by inhibiting phosphorylation on Ser 473 and Akt-mediated COX-2 induction. Inhibition of Akt phosphorylation and COX-2 expression may also cause Mcl-1 suppression and promote Bax translocation and activation. Because Akt also phosphorylates hexokinase (HK)-II and increases association with OMM through VDAC1 to inhibit cytochrome c release from mitochondria [33], Akt inhibition by embelin may cause release of HK-II from OMM and activation of VDAC1.

Interestingly, we found that the β-catenin level was potently decreased by embelin. β-Catenin expression is increased when GSK-3β is inactivated by phosphorylation on Ser 9 via Akt-dependent signals. Accumulation of β-catenin in the cytosol may cause nuclear translocation and activate transcription of downstream target genes such as cyclin D1, c-myc, and MMP-7. Our data showed that embelin treatment resulted in significant inhibition in the expression of β-catenin and prevents mRNA transcription of cyclin D1, c-myc, and MMP-7, which may be increased by the β-catenin-dependent pathway. Decreased levels of β-catenin may be caused by degradation of β-catenin because embelin strongly enhances GSK-3β-mediated phosphorylation of β-catenin on Ser 33/37 and Thr 41. The phosphorylated form of β-catenin is ubiquitinated by β-TrCP ubiquitin E3 ligase to be degraded by the proteasome [34]. Our data clearly showed that embelin may activate GSK-3β by inhibiting phosphorylation on Ser 9 and increases phosphorylation of β-catenin on Ser 33/37 and Thr 41 to induce ubiquitination of β-catenin by β-TrCP. Because it is suggested that Akt induces GSK-3β phosphorylation on Ser 9 to promote GSK-3β degradation through ubiquitin proteasome pathway, inhibition of Akt phosphorylation and activation by embelin partially contributes to the GSK-3β activation and inhibition in the level of β-catenin. Recently, Kim et al. have demonstrated that embelin suppresses the constitutive activation of the Akt/mTOR/S6K1 signaling cascade in human prostate cancer cells [28]. Thus, mTOR inhibition and Akt inhibition may be involved in the embelin modulation of β-catenin expression because mTOR also regulates the expression of β-catenin [35].

Additionally, embelin potently blocks prostate cancer cell migration and invasion. Our data also showed a significant decrease of MMP-2 activity in embelin-treated PC3 cells while MMP-9 activity was not significantly changed. Enhanced MMP-2 and MMP-9 activity has been found to contribute to cancer cell migration and invasion [36]. Because MMP-2 and MMP-9, as well as MMP-7, are known to be the downstream targets of β-catenin [37], significant inhibition in the level of β-catenin by embelin may inhibit MMP-2 activity and cancer cell migration. Recently, Huang et al. suggested that embelin inhibits epithelial-to-mesenchymal transition (EMT) by upregulating E-cadherin and inhibiting the expression of EMT-inducing transcription factors [38], including Snail, Slug, and ZEB1, and that this is correlated with MMPs inactivation in pancreatic cancer cells. Therefore, the possibility that embelin may suppress EMT to modulate MMP activity will need to be determined to understand the pro-apoptotic effects of embelin in prostate cancer cells.

In summary, our data show that embelin induces mitochondrial-dependent apoptosis by regulating the action of Bax and VDAC1 in human prostate cancer cells. Inhibition of Akt signaling and activation of GSK-3β partially contributes to the pro-apoptotic effect of embelin.
